# Frustration and its impact on search and rescue canines

**DOI:** 10.3389/fvets.2025.1546412

**Published:** 2025-03-07

**Authors:** Sally Dickinson, Erica N. Feuerbacher

**Affiliations:** Applied Animal Behavior and Welfare Lab, School of Animal Science, Virginia Polytechnic and State University (Virginia Tech), Blacksburg, VA, United States

**Keywords:** SAR dogs, frustration, stress, working dog welfare, HRV, wearable technology

## Abstract

Despite advances in modern technology, dogs remain the primary detection tool in search and rescue (SAR) missions, locating missing persons across diverse and dynamic environments, including wilderness, avalanche zones, water, and disaster areas. Their exceptional olfactory abilities, combined with their capacity to process complex discrimination tasks and adapt to varied environmental stimuli, make them uniquely suited for this work. However, SAR operations can be both physically and psychologically demanding, requiring sustained focus, endurance, and consistent performance under stressful conditions. Frustration, a form of psychological stress, arises when a dog encounters blocked access to a goal or when an expectation is violated, triggering physiological and behavioral changes that may impact performance. This study investigated the physiological and behavioral responses of SAR dogs to two distinct stress conditions: psychological stress induced by frustration and physiological stress induced by moderate exercise. We measured heart rate variability as an indicator of autonomic nervous system response to stress and analyzed search task performance to assess how frustration and exercise affected the dogs' latency and accuracy in executing their trained final response to the target odor. Our results revealed significant decreases in heart rate variability following frustration and increased latency in the search task, suggesting that frustration had a more pronounced impact on the dogs' physiological state and performance compared to exercise. By examining the effects of psychological and physiological stress, this study contributes to a deeper understanding of how different stressors influence SAR dog performance and welfare. These findings provide valuable insights for optimizing training methodologies and operational preparedness, ensuring both the effectiveness and well-being of SAR dogs in the field.

## 1 Introduction

Search and rescue dogs have played an integral role in the search for missing people, with records dating back to the 18th century, when the Monks of St. Bernard Hospice in the Swiss Alps trained dogs to aid in finding lost travelers (1). These dogs either led rescuers to the subject or assisted the subject to safety. During World War II, the Civil Air Defense Corps in Britain utilized dogs to locate survivors buried in the rubble of collapsed buildings after air raids. Dogs performed naturalistic behaviors such as digging at the rubble, whining or barking, and attracting the rescuers' attention to the survivors' location in the debris (2). As the twentieth century progressed, dogs were utilized in increasingly diverse and dynamic situations to detect a growing number of different odor targets, emphasizing the need for clear, trained responses to communicate successful detection of the specific target (3). In search and rescue (SAR) a specifically trained behavior facilitates clearer communication between handler and dog, helping prevent instances of dogs responding similarly to target stimuli and non-target stimuli (for example, scratching or digging to access food, animals, or dog toys) which could mislead handlers and result in the misallocation of search resources.

Today, SAR dogs must possess a range of desirable traits and behavioral repertoires in order to be successful in diverse and dynamic scenarios (4). Key attributes include persistence, adaptability, and the ability to maintain focus and respond accurately to trained cues (5). To develop these traits, handlers and trainers employ a variety of learning principles. One approach sometimes used to enhance persistence and motivation involves manipulating access to desired reinforcers, such as toys or other rewards. By temporarily restricting access to these reinforcers, trainers may induce the emotional response of frustration. This approach leverages frustration as a psychological motivator to elicit specific goal-directed behaviors, such as increased vocalization, navigating obstacles to reach a goal, stronger grip on a toy, and quicker, more forceful execution of a behavior.

Frustration, a psychological concept extensively discussed by Freud (6), was explored and incorporated into the frustration-aggression hypothesis by Dollard and Miller (7). Frustration arises when an individual's goals are blocked or an expectation is violated, often leading to heightened arousal and intensified goal-directed actions. These behavioral changes correlate with physiological responses driven by autonomic nervous system activation, such as increased heart rate and elevated stress hormone levels (8). Similar responses occur in dogs, where frustration manifests as heightened arousal, goal-directed behaviors, vocalization, and even displays of aggression. When the dog recognizes that the goal cannot be achieved, it may exhibit redirected behaviors, such as biting objects, or displacement activities, such as sniffing, scratching, yawning, and lip licking (9).

The physiological response to frustration follows the same process as other psychological and physiological stress-inducing stimuli (10). Stress is the body's evolutionary beneficial physiological response to environmental stimuli which threaten an individual's ability to maintain homeostasis. When an organism perceives a threat or encounters obstacles to achieve its goals, the autonomic nervous system activates the hypothalamic-pituitary-adrenal (HPA) axis and the sympathetic-adreno-medullary (SAM) axes (11). The HPA and the SAM axis release glucocorticoid hormone (primarily cortisol) and catecholamines (epinephrine and norepinephrine) respectively. Cortisol increases available glucose in the bloodstream, while epinephrine and norepinephrine rapidly increase heart rate and blood pressure, preparing the individual to take action to mitigate the threat or overcome the obstacle. Stress can arise from physical demands (such as prolonged motor activity) and psychological challenges (such as blocked access to a desired goal). The consequences of prolonged or intense frustration can contribute to chronic stress in SAR dogs, which is linked to a variety of behavioral and health concerns in dogs (9).

An emerging non-invasive, real-time tool for assessing stress is heart rate variability (HRV) (12). HRV studies in humans have shown HRV to be a reliable predictor of an individual's response to a psychological stressor and a biomarker of a psychological stress response (12). Studies in dogs have also documented correlations between HRV and behavioral responses. For example, Craig et al. found significant negative correlations between HRV and aggression in dogs (13), while Katayama et al. found that HRV was a positive predictor of emotional states in positive and negative situations (14). HRV refers to the variation in time intervals between heartbeats which is influenced by the autonomic nervous system. Generally, a higher HRV indicates a relaxed state with balanced autonomic control, while a lower HRV often reflects heightened sympathetic nervous system activity, characteristic of a stress response (15). Physiological stress typically results in a predictable decrease in HRV and a quick return to baseline or increase in HRV as the body returns to homeostasis. In contrast, Psychological stress, such as frustration, can produce a prolonged and variable decreases in HRV as it is influenced by cognitive and emotional processes that sustain the sympathetic nervous system activation (16).

Research indicates that stress, whether stemming from physical or psychological sources, can adversely affect a dog's performance, impacting both welfare (17) and task accuracy (18). Elevated stress levels may trigger physiological and behavioral changes that disrupt a search and rescue (SAR) dog's detection ability, leading to increased errors or delayed responses (4). However, it remains unclear whether different types and intensities of stressors, such as frustration versus physical exertion, produce distinct effects on SAR dogs.

We utilized a within-subject design to investigate how psychological stress (frustration) and physiological stress (physical exertion) influence the welfare and detection performance of SAR dogs. We measured HR, HRV, and behavioral responses of the dogs during a baseline condition, frustration condition, and physical exertion condition. We also assessed dogs' latency to detect a target odor and accuracy during a search task, which dogs completed after each of the three conditions. Handlers also completed the Canine Frustration Questionnaire (9) to allow us to assess how the participant dogs compared to other dogs on the factors identified in the questionnaire. By examining physiological and behavioral responses to these stressors, we sought to provide insights that could guide handlers in developing more effective and humane training protocols.

## 2 Materials and methods

**Figure 1:**
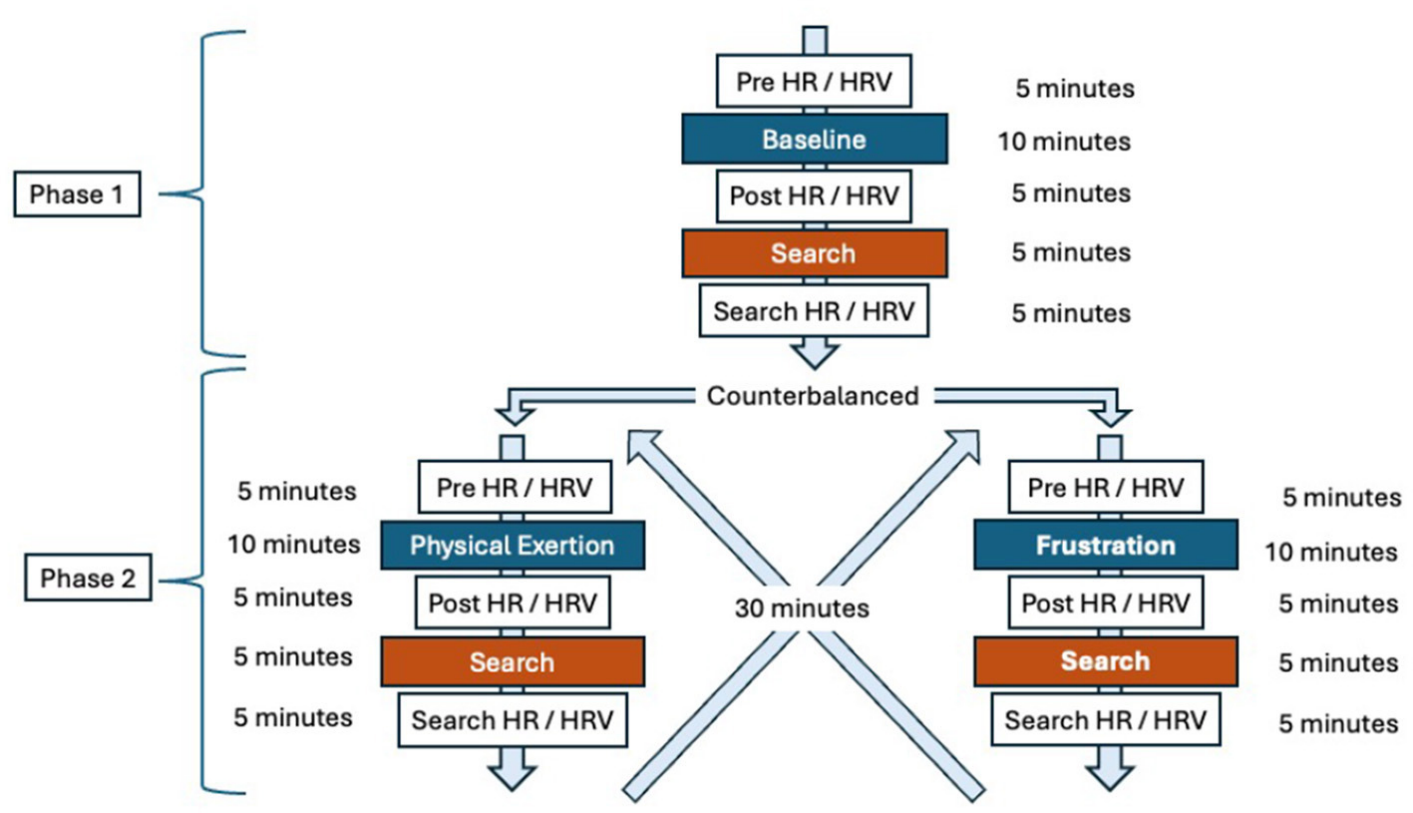
Overview and flow of the study design. The study consisted of two phases: baseline (Phase 1), frustration, and physical exertion (Phase 2, counterbalanced). Each phase was followed by a search task. Heart rate (HR) and heart rate variability (HRV) were measured after each phase and after each search. This figure provides a visual summary of the experimental layout and procedural sequence.

Approval to conduct this study was granted by the Virginia Tech Institutional Animal Care and Use Committee (IACUC #23-136) and the Virginia Department of Emergency Management, Chief of Special Operations. The study consisted of two phases, each followed by a search task. Phase 1 served as the baseline condition (no induced stress), while Phase 2 alternated between the frustration condition and the physical exertion condition, counterbalanced across subjects to minimize order effects. Physiological data, including heart rate (HR) and heart rate variability (HRV), were collected before and after each condition and again following the corresponding search task. Behavioral data, such as the latency to perform the trained final response and search accuracy, were recorded during each search. Figure 1 provides a detailed overview of the study's layout and procedural flow.

### 2.1 Participants

We recruited participants from the Virginia Search and Rescue (SAR) canine community, meeting the selection criteria for operational or in training for canine team status as defined under the Virginia Department of Emergency Management (VDEM) SAR program standards. Participants specialized in either live find area search or human remains area search. Recruitment was conducted via an email invitation sent to all canine handlers in the Commonwealth of Virginia, with final selection based on availability on testing dates.

### 2.2 Canine frustration questionnaire

Before the day of the experiment, participants completed the Canine Frustration Questionnaire (9) using an online survey platform. At the beginning of the questionnaire, handlers provided the following details: The name of the dog for which the questionnaire was completed, the dog's certified discipline, the handler's name. Additional questions included: the handler's perception of the dog's drive level (low, medium, or high), the types of collars the dog had ever worn during training or operational work (e.g., GPS collar, electronic training (e-collar), pinch collar, flat collar), the dog's age and sex. The full questionnaire is available in the Supplementary material.

#### 2.2.1 Study area layout

**Figure 2:**
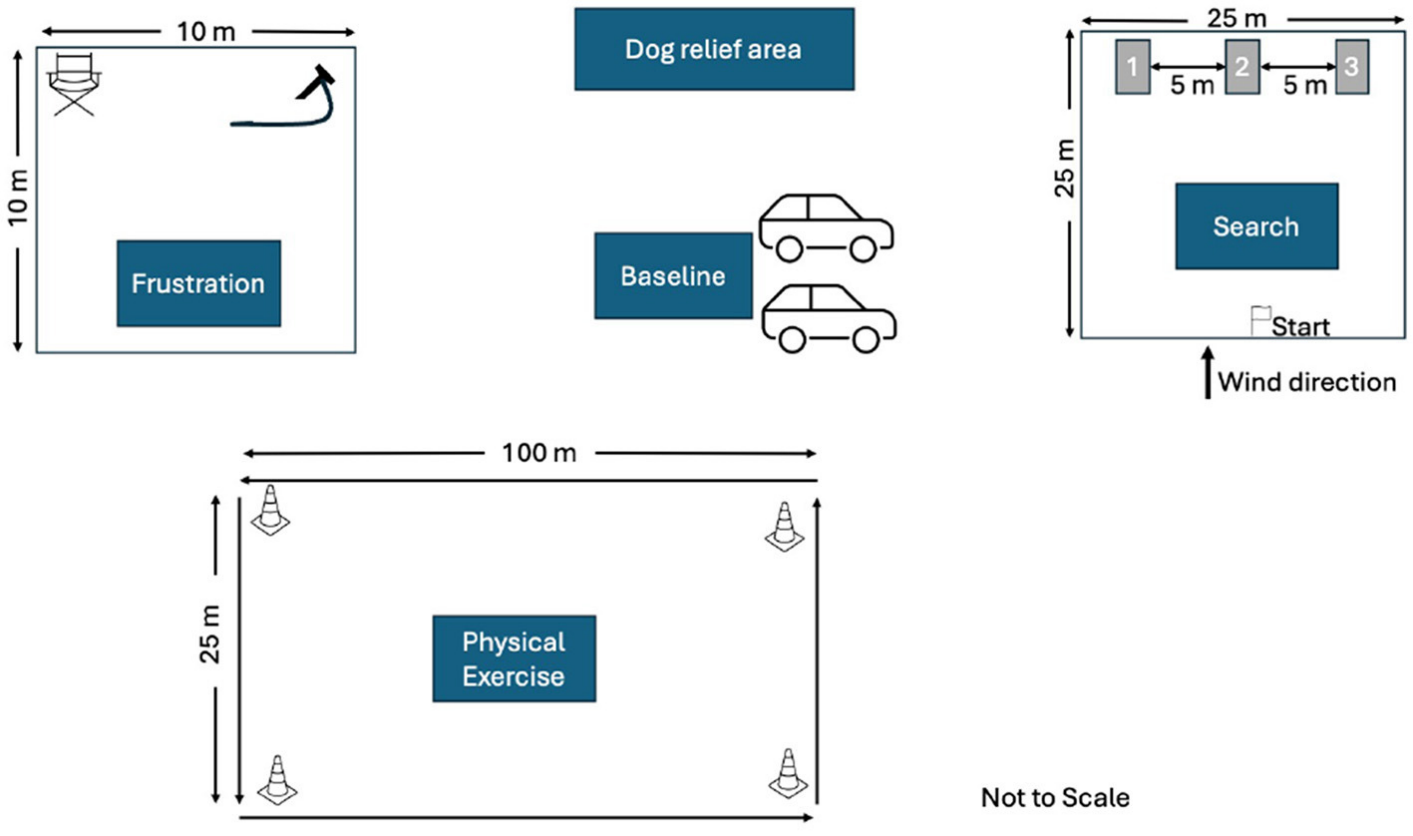
Typical layout of study spaces. Specific areas were designated for parking, dog bathroom, frustration condition, physical exertion condition, and the search area. Adjustments were made as needed to accommodate site differences.

We conducted the experiment at four different outdoor locations (three in Virginia and one in Maryland) to facilitate access by the SAR dog teams. The testing areas included three mowed grass fields and one empty gravel parking lot. We instructed the handlers to follow their normal routines for taking their dogs to a typical SAR training event. At each site we designated a parking area, a dog bathroom area, frustration condition area, exercise condition area and the search area. Figure 2 shows the typical layout of the study spaces, though slight adjustments were made to accommodate individual site differences.

For the physical exertion condition, we created a rectangular course measuring 100 m × 25 m, ensured it was free of trip hazards, and marked the corners with cones to guide the handler and dog's path. Before starting this condition, handlers were provided with a link to download a metronome app on their phones to help maintain a consistent walking pace. The frustration condition area was a 10 m × 10 m space, with a snow fence perimeter to obstruct the dog's view. Inside the area, we placed a camp chair and a 1 m tie-out lead securely attached to the ground via a stake. Before starting the frustration condition, the dog's usual reward toy, five additional toys, and any handler equipment (e.g., a working dog vest) were placed in a container outside the perimeter fence, out of the dog's sight. The search area was marked as a 25 m × 25 m square, with cones at each corner. An additional cone was placed at the midpoint of the windward side to designate the starting point. On the opposite side of the square, three visually identical bark-barrels were positioned. Bark-barrels are constructed by securing two 55-gallon barrels together, with the bottom removed from one barrel and attached to the top of the other to elongate the structure. Each bark-barrel was equipped with a removable door held in place by an internal bungee. Bark-barrels are used in detection dog training to conceal a human subject or another target odor. The barrels were numbered 1, 2, and 3 from left to right. For each search task, the target odor was assigned to one of the bark-barrels using a dice roll to ensure randomization. The remaining two barrels were kept odor-free. The position of the target odor was kept blind to the handler. A GoPro camera was positioned to record the dog's search behavior around the bark-barrels.

### 2.3 Procedures

#### 2.3.1 Physiological monitoring

We assigned handlers specific arrival times at the study site. Upon arrival, we instructed handlers to remove all collars from their dogs, except for a flat collar. We then fitted a PetPace 2.0 collar (19) above the flat collar, closer to the dog's head, following the manufacturer's guidelines for tightness and position. The PetPace collar remained on the dog throughout all study conditions, and handlers were instructed to use only the flat collar for attaching a leash to prevent interference with the collar. After the initial fitting, the PetPace web portal was monitored to confirm data recording. Once data became available, the reported heart rate was manually verified by palpating the femoral or brachial artery. If discrepancies were detected, the collar's position or fit was adjusted until the reported values were consistent for five consecutive minutes. If the collar failed to report data during any monitoring period, it was readjusted until data points were recorded and manually verified via pulse check. During designated times for heart rate (HR) and heart rate variability (HRV) monitoring (pre- and post-condition, as well as post-search), handlers were instructed to position their dogs in a down position at their side and remain quietly standing. This procedure ensured that the acoustic sensors accurately isolated the dog's heart rate, minimizing interference from movement. For the 5-minute monitoring periods, approximately 1 min was allotted for the handler to position the dog, followed by recording the next two values reported by the PetPace collar, which transmits data every 2 min. When not actively engaged in the study, dogs were housed in their regular crates inside their handlers' vehicles, consistent with typical SAR training practices. While transitioning between conditions, searches, or returning to the vehicle, dogs were kept on a leash. Before beginning any study activities, handlers were instructed to provide their dogs with a bathroom break in a designated area. During study activities, dogs were either leashed or off-leash under their handlers' verbal control, depending on the specific requirements of the study component.

#### 2.3.2 Conditions

All dogs begin in the baseline condition and then were counterbalanced between frustration and physical exertion. Each condition, including all monitoring and search took 30 minutes. Between conditions the dogs were in their regular crates in their vehicles Figure 1.

##### 2.3.2.1 Baseline condition

The baseline (BL) condition took place next to the handler's vehicle. To minimize distractions, no other dogs were in sight, and there was no activity in the vicinity. The PetPace collar had already been fitted and verified to be transmitting data. The baseline condition began with the handler placing their dog in a down position next to them for a 5-minute monitoring period (pre-BL monitoring). Following this, the handler waited quietly with their dog for 10 minutes, simulating the experience of waiting their turn during SAR training. During this time, the dog was allowed to remain in whatever position was most comfortable—sitting, standing, or lying down. After the 10-minute baseline activity, the handler placed the dog in a down position again for another 5-minute monitoring period (post-BL monitoring). After the post-BL monitoring was complete, the handler walked the dog directly to the search area to complete the search. After the search, the handler placed the dog in a down position again for another 5-minute monitoring period (post-search monitoring) then returned the dog to their vehicle.

##### 2.3.2.2 Frustration condition

The frustration condition (FC) was divided into three components based on the “Behavioral and Physiological Correlates of the Canine Frustration Questionnaire” (20): changed contingency; blocked access; and withdrawn attention. All the dogs experienced all three components in the same order. These components were selected as the most impactful for a SAR dog. During all the components of the FC the researcher, handler and dog remained inside the 10 m × 10 m fenced area. The researcher remained in a corner away from the dog and did not interact with the dog except as described in changed contingency component. Before beginning the FC, the handler entered the area with the dog on a lead and positioned the dog in a down for the pre-FC 5-minute monitoring period. Following this, the handler attached the dog's collar to the tie-out lead and removed their own lead to start the first component, changed contingency. Once the dog was secured to the tie-out, the researcher entered the area with a container of toys and positioned themselves just out of the dog's reach. To engage the dog, the researcher called the dog's name and, depending on the type of toy, either slapped it against their hand, bounced it, or shook it. Beginning with the dog's usual reward toy, the researcher teased the dog with each toy for approximately 5 s before placing it 25 cm out of the dog's reach. This process was repeated for all five toys. After the last toy was placed, the toys were left in place for 30 s before the researcher collected them in reverse order, placed them back in the container, and removed them from the area. During this component, the handler remained stationary and quiet, positioned 2 meters behind the dog. Once the toys were collected, the handler reattached their lead, unhooked the tie-out, and avoided interacting with the dog in any other way. The second component, blocked access, began immediately afterward. The researcher handed the handler any equipment the dog typically wore for SAR work and instructed the handler to prepare the dog as they would for a search by placing the working vest or collar on the dog. The handler then gave the search cue but did not release the dog. If the dog pulled against the leash, the handler remained quiet, still, and stationary. After 1 min, the handler removed the equipment as they normally would, without speaking or otherwise interacting with the dog. The final component, withdrawn attention, started as soon as the search equipment was removed. The researcher directed the handler to a chair and instructed them to release the dog from the lead once seated. The handler remained seated for 5 minutes and was instructed not to look at, speak to, or touch the dog during this time. At the end of the withdrawn attention component, the handler called their dog and placed them back on the leash. The total duration of the FC was 10 min, which was directly followed by the 5-min post-FC monitoring period. After the post-FC monitoring was complete, the handler walked the dog directly to the search area to complete the search. After the search, the handler placed the dog in a down position again for another 5-minute monitoring period (post-search monitoring) then returned the dog to their vehicle.

##### 2.3.2.3 Physical exertion condition

The handler, with the dog on a lead, approached the physical exertion (PE) area and was instructed to position the dog in a down for the 5-minute pre-PE monitoring period. The handler was directed to set the metronome app on their phone to 130 beats per minute (bpm). Following this, the handler, with the dog on a lead, walked the perimeter of the PE course, matching each footfall to the beat of the metronome. This established a walking pace between 6.5 and 8 km/h. Handlers were instructed not to place their dogs under specific obedience cues, such as “heel,” but to simply walk with the dog. They were encouraged to verbally motivate their dogs if they slowed below the target pace and to gently slow them down if they went too fast. The total duration of the PE condition was 10 minutes. Upon completion, the handler placed the dog in a down position for the 5-minute post-PE monitoring period. After the post-PE monitoring was complete, the handler walked the dog directly to the search area to complete the search. After the search, the handler placed the dog in a down position again for another 5-minute monitoring period (post-search monitoring) then returned the dog to their vehicle.

#### 2.3.3 Search task

Prior to the first search task, we asked the handler to describe the dog's behaviors that indicated recognition of its target odor. We also requested a detailed description (topography) of the trained final response (TFR). For each search, we instructed the handler to signal with a thumbs-up when they believed the dog had identified the correct bark-barrel based on their interpretation of the dog's behavior. After completing the 5-minute post-condition monitoring period (BL, FC, or PE), the handler, with the dog on a lead, proceeded directly to the search area to perform the search task. Following each search, the dog received its reward toy, and the handler engaged in play according to the dog's usual reward routine for 2 minutes before placing the dog in a down position for the 5-min post-search monitoring period. The handler and dog approached the search area with the dog on a lead. We instructed the handler to prepare the dog as they would for an odor recognition trial, give the search cue, and release the dog. After giving the cue, the handler remained at the designated start location in silence, except to recall the dog if it left the search task area. Once released, the dog had 5 minutes to approach and investigate any or all of the three bark-barrels to identify the one containing its target odor. Using the handlers' descriptions of their dogs' behavior and their indication the dog was at the correct target, we measured the latency of the TFR after the dog arrived at the correct bark-barrel. For dogs that exhibited an almost immediate response, we recorded a minimum latency of 0 second. If a dog performed its TFR at the incorrect bark-barrel we immediately informed the handler that the response was incorrect. The handler, following prior instructions, responded as they would during a standard training session to communicate this to the dog. We recorded the number of incorrect responses (false negative) for each search.

#### 2.3.4 Analysis

During each 5-min monitoring period, we recorded two data points from the PetPace collar. For all analyses, we used the second data point to ensure readings were less affected by movement or heavy respiration. For each condition (baseline, frustration, and exercise) we calculated the change in HR and HRV by subtracting the pre-condition values from the post-condition values (Post-Pre) to assess the physiological effects of each condition. Additionally, we subtracted the pre-condition values from the post-search values (Search-Pre) to evaluate the combined effects of the condition and the search. We analyzed these results using a one-way ANOVA with a Tukey *post hoc* test for pairwise comparisons. HRV is reported as vagal tone by the PetPace system, with the calculation method being proprietary and not available for review. All HRV analyses utilized the reported values directly from the PetPace system without any additional manipulation. Additionally, we reported the results of the Canine Frustration Questionnaire, as well as the latency and false negative outcomes from the search tasks.

### 2.4 Results

#### 2.4.1 Participants

We recruited 23 handler-dog teams via an email invitation sent to the Commonwealth of Virginia SAR community. Of these, 12 teams were available for testing. One dog, trained exclusively with food, was excluded from the study because the frustration condition was designed around blocked access to a toy. Consequently, 11 dogs completed all three conditions, and their handlers completed the Canine Frustration Questionnaire. Table 1 provides the age, breed, and SAR discipline of the participating dogs. Seven dogs were identified as high drive, three as medium drive, and one as low drive. All but one dog had experience wearing a GPS collar, and all but four had worn either a pinch collar or an e-collar.

**Table 1:** Demographics and disciplines of the participating dogs.

**Dog name**	**Breed**	**Age**	**Sex**	**Discipline**
Zöe	Border Collie	7	FS	HRD
Reagan	Belgian Tervuren	5	FS	LF
Loki	Border Collie	11	MN	LF
Memphis	German Shepherd	2	F	LF
Val	Belgian Malinois	5	FS	HRD
Ginja	Australian Shepherd	3	FS	HRD
Fenn	Labrador Retriever	2	F	LF
Joker	German Shepherd x Malinois	7	MN	HRD
Vader	Labrador Retriever	6	MN	LF
Timber	Labrador x Hound	2	MN	LF
Moose	Labrador Retriever	3	M	LF

SAR disciplines include Live Find (LF) and Human Remains Detection (HRD). Sex is denoted as Female Spayed (FS), Male Neutered (MN), Female (F), and Male (M).

#### 2.4.2 Canine frustration questionnaire

All handlers completed the Canine Frustration Questionnaire prior to the day of the study. Table 2 shows the normal ranges for each of the principal components, the individual dogs' results, and the mean with standard deviation for the group. The Canine Frustration Questionnaire (CFQ) scores were calculated using the scoring methodology outlined in McPeake et al. (9).

**Table 2:** Canine frustration questionnaire results.

**Dog**	**Overall CFQ (norm 0.33–0.57)**	**PC1 general frustration (norm 0.23–0.53)**	**PC2 barrier frustration and perseverance (norm 0.37–0.73)**	**PC3 unmet expectations (norm 0.35–0.69)**	**PC4 autonomous control (norm 0.24–0.50)**	**PC5 frustration coping (norm 0.30–0.62)**
Zöe	0.43	0.36	0.55	0.65	0.25	0.40
Reagan	0.39	0.28	0.45	0.45	0.30	0.33
Loki	0.49	0.32	0.55	0.70	0.35	0.53
Memphis	0.56	0.48	0.85	0.65	0.30	0.53
Val	0.57	0.48	0.75	0.80	0.30	0.53
Ginja	0.66	0.56	0.55	0.90	0.70	0.47
Fenn	0.81	0.84	0.90	1.0	0.50	0.87
Joker	0.44	0.44	0.35	0.55	0.25	0.47
Vader	0.42	0.24	0.45	0.65	0.40	0.40
Timber	0.50	0.48	0.65	0.80	0.25	0.33
Moose	0.38	0.44	0.40	0.20	0.25	0.67
**Mean**	**0.51**	**0.45**	**0.59**	**0.67**	**0.35**	**0.50**
**±Std. Dev**.	**0.13**	**0.16**	**0.18**	**0.22**	**0.14**	**0.16**

The table shows the five principal components identified in the questionnaire (normal ranges for each principal component are in parentheses under the title). Individual dog scores, and the group's mean and standard deviation are reported (bold).

#### 2.4.3 Physiological monitoring

**Figure 3:**
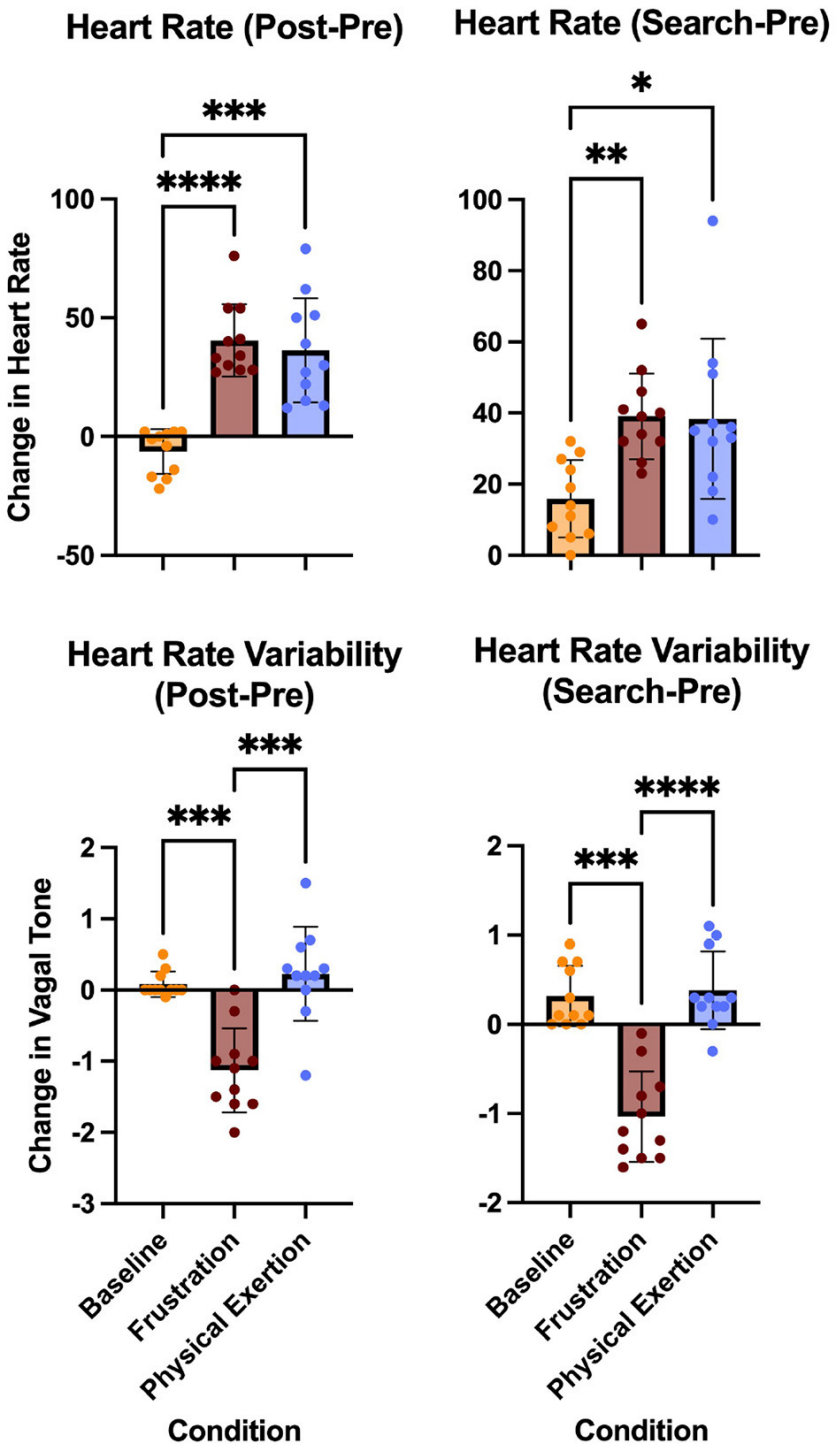
Changes in heart rate and heart rate variability across conditions (baseline, frustration, and physical exertion). Post-Pre values represent the difference between post-condition and pre-condition monitoring, while Search-Pre values represent the difference between post-search and pre-condition monitoring. Significant differences in heart rate and heart rate variability changes are indicated by * (*P* < 0.05), ** (*P* < 0.01), *** (*P* < 0.001), and **** (*P* ≤ 0.0001) for specific condition comparisons, including baseline to frustration, baseline to physical exertion, and frustration to physical exertion.

The following HR and HRV results refer to Figure 3 which illustrates the change (post-pre and search-pre) across conditions for HR and HRV. For all, *n* = 11.

##### 2.4.3.1 Heart rate post-pre change

The mean values and standard deviation for the conditions were; Baseline (M = -6.273, SD = 9.435), Frustration (M = 40.45, SD = 15.26) Physical exertion (M = 36.36, SD = 21.94). A one-way ANOVA revealed a significant effect of conditions on heart rate, F(1.953, 1953) = 29.28, *p* < 0.0001, η^2^= 0.7454. Tukey's HSD *post hoc* test showed that both the frustration condition and the physical exertion condition post-pre change were significantly greater than baseline (*p* < 0.0001, and *p* = 0.0002, respectively). Tukey's HSD *post hoc* test showed that the physical exertion condition was not significantly different from the frustration condition (*p* = 0.8423).

##### 2.4.3.2 Heart rate search-pre change

The mean values and standard deviation for the conditions were; Baseline (M = 15.91, SD = 10.90), Frustration (M = 39.09, SD = 12.03) Physical exertion (M = 38.36, SD = 22.53). A one-way ANOVA revealed a significant effect of conditions on heart rate, F(1.622, 16.22) = 9.184, *p* = 0.0033, η^2^ = 0.3078. Tukey's HSD *post hoc* test showed that both the frustration condition and the physical exertion condition search-pre change were significantly greater than baseline (*p* = 0.0010, and *p* = 0.0209, respectively). Tukey's HSD *post hoc* test showed that the physical exertion condition was not significantly different from the frustration condition (*p* = 0.9938).

##### 2.4.3.3 Heart rate variability post-pre change

The mean values and standard deviation for the conditions were; Baseline (M = 0.0818, SD = 0.1779), Frustration (M = -1.127, SD = 0.5884) Physical exertion (M = 0.2273, SD = 0.6604). A one-way ANOVA revealed a significant effect of conditions on heart rate variability, F(1.997, 19.97) = 28.96, *p* < 0.0001, η^2^ = 0.7433. Tukey's HSD *post hoc* test showed that the frustration condition change was significantly lower than baseline (*p* = 0.0002) and the frustration condition change was significantly lower than the physical exertion condition (*p* = 0.0001). Tukey's HSD *post hoc* test showed that the physical exertion condition was not significantly different from baseline (*p* = 0.7451).

##### 2.4.3.4 Heart rate variability search-pre change

The mean values and standard deviation for the conditions were; Baseline (M = 0.3182, SD = 0.3401), Frustration (M = -1.036, SD = 0.5065) Physical exertion (M = 0.3818, SD = 0.4355). A one-way ANOVA revealed a significant effect of conditions on heart rate variability, F(1.692, 16.92) = 34.46, *p* < 0.0001, η^2^ = 0.7751. Tukey's HSD *post hoc* test showed that the frustration condition change was significantly lower than baseline (*p* = 0.0003) and the frustration condition was significantly lower than the physical exertion condition (*p* < 0.0001). Tukey's HSD *post hoc* test showed that the physical exertion condition was not significantly different from baseline (*p* = 0.9082).

#### 2.4.4 Behavioral responses

##### 2.4.4.1 Latency

**Figure 4:**
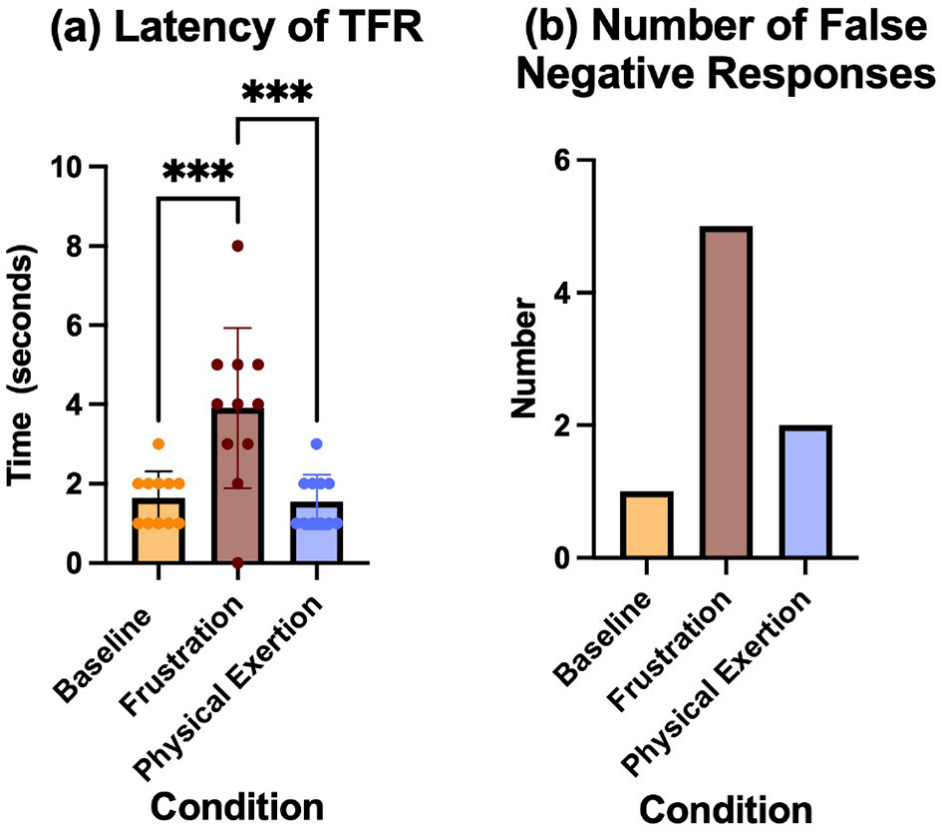
**(A)** Latency to perform the trained final response at the correct bark-barrel, shown with mean and standard deviation for each condition:baseline, frustration and physical exertion. *** Indicates *p* < 0.001. **(B)** The total number of false negatives recorded during searches for each condition.

*n* = 11. The mean values and standard deviation for the conditions were; Baseline (M = 1.636, SD = 0.6742), Frustration (M = 3.909, SD = 2.023) Physical exertion (M = 1.545, SD = 0.6876). A one-way ANOVA revealed a significant effect of conditions on latency, F(2, 30) = 11.79, *p* = 0.0002, η^2^ = 0.4402. Tukey's HSD *post hoc* test showed that the frustration condition change was significantly greater than baseline (*p* = 0.0008 and significantly greater than the physical exertion condition (*p* < 0.0005). Tukey's HSD *post hoc* test showed that the physical exertion condition was not significantly different from baseline (*p* = 0.9851) (Figure 4A).

##### 2.4.4.2 False negative

*n* = 11. The total number of false negatives in the search after each conditions was baseline = 1, frustration = 5, physical exertion = 2 (Figure 4B).

## 3 Discussion

Handlers may intentionally use frustration during training to habituate dogs to challenging conditions they might encounter in real-world searches or to elicit specific desired responses, such as increased intensity of effort. However, there is limited research on whether frustration in training impacts SAR dogs' performance and welfare. This study investigated whether different types of stressors, specifically frustration and physical exertion, produce distinct effects on SAR dogs' welfare and detection performance. Among the sample population of 11 SAR dogs, we observed that these stressors had a differential effects on heart rate variability but not on heart rate. Additionally, the increased latency to perform the trained final response at the target and the highest number of instances of incorrect responses (false negatives) were associated with the frustration condition. Our results suggest that inducing frustration in SAR dogs can negatively impact both their welfare and performance, and its use as a training tool should be carefully evaluated.

### 3.1 Canine frustration questionnaire (CFQ)

The results of the Canine Frustration Questionnaire (CFQ) revealed that the SAR dogs evaluated in this study fell within the typical range for companion dogs across all five principal components (9). To our knowledge, this is the first direct comparison of CFQ scores between SAR dogs and the companion dog population reported by McPeake. The CFQ principal components are defined as general frustration (PC1), barrier frustration (PC2), unmet expectations (PC3), autonomous control (PC4), and frustration coping (PC5), each of which relates to specific aspects of a dog's environment and their behavioral responses (20). Higher-than-normal scores in these components may indicate that a dog is struggling to cope with the particular environmental scenario (20). Search and rescue dogs are often considered distinct from companion dogs due to their rigorous selection processes and specialized training (21–23). However, our findings suggest that the SAR dogs in this study are behaviorally similar to companion dogs in their overall frustration levels. Of particular relevance to SAR work are PC2 (barrier frustration) and PC5 (frustration coping), as these components address scenarios commonly encountered by SAR dogs: encountering obstacles that impede goal achievement and coping with frustrating circumstances, suggesting lower scores in these components would be desirable for SAR dogs. It is generally accepted that one or more of the following training principles be used to develop frustration tolerance; desensitization (successive exposures to increasingly frustrating experiences) and counterconditioning (providing a positively valanced experience or item with the frustrating experience) (24). Typical SAR training that encourages a dog to hunt for longer periods of time, traverse increasingly more difficult terrains and work for longer without a reward all end with a reward (3). This has the effect of building frustration tolerance into SAR training, which would suggest that SAR dogs should have lower than general population levels of frustration. In this study, the mean scores for SAR dogs across all CFQ components fell within the normal ranges reported by McPeake's analysis of 2,346 companion dogs. This suggests that, despite their unique experiences as SAR dogs, the sample population in this study demonstrates frustration-related behaviors and coping strategies comparable to those of companion dogs.

### 3.2 Physiological monitoring

We found that SAR dogs had a significant increase in HR and a significant decrease in HRV after the frustration condition. Conversely, we found only a significant increase in change HR after the physical exertion condition. This suggests that the frustration condition had a differential effect on the dogs' physiological state compared to physical exertion. The increased HR and decreased HRV after frustration together indicate that inducing frustration can have a negative impact on the dogs' welfare. Physiological measures of heart rate (HR) and heart rate variability (HRV) have become critical components of monitoring human well-being, particularly with the advent of wearable technology (25). HRV, in particular, is a valuable indicator of the body's ability to mitigate future stress events and of its reaction to experienced stressors (26). In dogs and other animals, HRV has been shown to positively correlate with emotional states, providing insight into their stress and welfare status (14, 15). We used the PetPace collar, which employs a proprietary algorithm to analyze HRV and reports the results as a vagal tone index. Vagal tone index is a non-linear measure of parasympathetic nervous system (PNS) activity, specifically reflecting the vagus nerve's influence on heart rate. It indicates the PNS's ability to slow the heart rate and help the body return to homeostasis. A higher vagal tone index suggests better autonomic regulation and is considered an indicator of stress resilience (27). Despite its utility, there is limited data on HRV in dogs, and the variety of methods used to report HRV–including proprietary algorithms, time-domain, and frequency-domain measures–complicates cross-study comparisons. For this reason, we focused on changes in HR and HRV rather than absolute values in our analysis. As expected, HR significantly increased from pre- to post-condition and pre- to post-search in both the frustration and physical exertion conditions compared to baseline. This aligns with the activation of the autonomic nervous system in response to stress or physical activity, where elevated HR reflects heightened arousal or energy expenditure. These findings are consistent with prior research showing that both psychological stressors, such as frustration, and physical exertion stimulate a physiological response characterized by increased HR. In contrast, HRV showed distinct patterns: it significantly decreased from baseline to frustration, reflecting the physiological impact of short-term stress or fatigue. This finding aligns with previous research in both humans and animals, where decreased HRV is a marker of acute stress, driven by diminished parasympathetic activity and increased sympathetic dominance (27). The large effect sizes observed across the four physiological metrics (HR Post-Pre, HR Search-Pre, HRV Post-Pre, and HRV Search-Pre) underscore the substantial impact of frustration on SAR dogs' HRV, suggesting that even short-term psychological stressors can significantly alter autonomic regulation. To minimize interference, physiological data were collected only during stationary periods. The PetPace collar, designed with acoustic-based technology that does not require skin contact, was particularly suitable for canine use. However, it reliably reports data only when the dog is stationary and not panting. To address this limitation, dogs were placed in a down position during monitoring, ensuring at least one valid data point was recorded for each dog before and after each condition and search. Future studies should aim for near-constant monitoring throughout condition changes to better capture the full spectrum of physiological responses. Continuous data would allow for analyses of both short-term and long-term HRV trends. A potential confounding factor in studies using wearable technology is the effect of the device itself. Based on the dogs' prior histories of wearing GPS and e-collars, we do not anticipate that the PetPace collar influenced their physiological or behavioral responses. If the collar had acted as a stressor, we would have expected elevated HR and reduced HRV during the baseline condition. Additionally, while dogs with prior e-collar training might associate tight, heavier collars with aversive stimulation, no such differential responses were observed. Given the within-subject design of this study, changes in HR and HRV were evaluated across conditions, allowing us to isolate the effects of the conditions themselves.

### 3.3 Behavioral responses

In this study, we challenged the dogs with a three-object search task after each condition. This type of task, commonly referred to as an odor recognition test, is a fundamental component of detection dog training. The results revealed that, following the frustration condition, the dogs exhibited significantly longer latencies to respond to the target odor and a higher number of false negative responses. Specifically, the frustration condition resulted in five false negatives, compared to just one in the baseline condition and two in the physical exertion condition. In human research, increased latency has been linked to mild frustration events (28), although the precise mechanisms underlying this latency are not fully understood. The relationship between increased latency and false negatives in detection work with dogs remains unexplored. In dogs, task accuracy has been associated with frustration (29). Furthermore, communicative and affiliative behavior was found to decrease toward the handler in periods of frustration (30). Despite the minimal level of frustration inflicted during the frustration condition, which was designed to mirror mild training scenarios, the performance impact was evident. None of the dogs displayed aggressive behaviors or extreme responses indicative of an exaggerated stress reaction. However, the measurable effects on search performance suggest that even mild frustration can detrimentally influence a SAR dog's efficiency. A SAR dog's reaction to the trained target odor is its most critical behavioral response (3). Delays in initiating the TFR can allow the dog to become distracted, potentially leading to missed opportunities to effectively communicate a find. Incorrect responses, such as false negatives (when the target is present but the dog selects the incorrect location) or false positives (when no target is present but the dog signals an alternate odor), are equally problematic. Both errors have significant operational implications: they can mislead responders and result in the misallocation of resources, which could ultimately delay a rescue or recovery effort. Furthermore, in training contexts, incorrect responses often result in aversive corrections from the handler or, at minimum, a reduction in reinforcement opportunities. While these corrections are intended to guide the dog toward the desired behavior, they may inadvertently exacerbate performance issues (31) if the underlying cause of the error—such as frustration—is not addressed. By taking into account the activities a dog was engaged in prior to a task and recognizing the potential for reduced accuracy following stress events like frustration, handlers can structure training sessions to maximize the dog's likelihood of success.

Our findings emphasize the importance of balancing training techniques that incorporate frustration with considerations for the dog's psychological well-being and operational performance. Operationally SAR dogs are frequently exposed to frustration-inducing scenarios, such as barriers, obstacles, delays, or unpredictable reinforcement. While tolerance for such conditions is crucial for their work, the methods used to train this require careful evaluation. Our results indicate that inducing frustration negatively impacts both welfare and performance. Future research should investigate alternative training approaches that prepare dogs to perform effectively in challenging SAR environments without explicitly inducing frustration–or by applying it only at minimal levels to avoid adverse effects.

### 3.4 Limitations

In our study, we used the Canine Frustration Questionnaire. However, survey data are often subject to bias (32). Given the close working relationships handlers have with their SAR dogs, it is reasonable to expect that handlers may under-report problem behaviors and over-report desirable behaviors. Future research should investigate whether the trends in overall frustration observed in this study population of SAR dogs align with those of SAR dogs from different regions or operational contexts. Expanding the scope of survey data would help to clarify whether the patterns observed here are representative of SAR dogs more broadly. In our experiments on the physiological and behavioral impacts of frustration and physical exertion, we enrolled 11 SAR dogs. While this sample size was sufficient to detect significant effects of the stressors examined, replication with a larger and more diverse sample is necessary to fully understand the impact of psychological stressors on SAR dogs. Although these dogs had a range of prior learning histories, it is possible that stressors might have different effects on dogs with different training backgrounds or experiences. Thus, additional studies evaluating these parameters in different study populations would be useful to understand the generality of our results. A further limitation of this study lies in the technology used. The PetPace collar, while user-friendly and robust enough to withstand the demands of SAR training, has notable constraints. Its data recording is sporadic and restricted to periods when the dog is stationary, which limits the ability to monitor physiological responses during active conditions. To build on this work, we recommend exploring alternative technologies, such as the Polar H10 human heart rate monitor (Polar Electro, 2024)^1^, provided an appropriate strap can be developed to avoid interfering with the dog's natural movement while maintaining optimal positioning. Furthermore, devices that transmit raw data would be highly advantageous, as they would enable independent analysis and facilitate direct comparisons with other research findings.

### 3.5 Conclusion

Despite its limitations, this study provides valuable insights into the effects of psychological and physical stressors on SAR dogs. These findings highlight the need for further research to better understand the impact of stressors and to develop effective methods for mitigating their effects. The preliminary results demonstrate that physiological and behavioral responses in SAR dogs differ depending on the type of stressor encountered. While these changes may not be consistent across all SAR dogs or stressors, handlers should be mindful of these potential effects and design training protocols that prioritize the dogs' welfare while fostering successful learning experiences.

## Data Availability

The raw data supporting the conclusions of this article will be made available by the authors, without undue reservation.

## Footnotes

^1^https://www.polar.com/us-en/ (accessed June 5, 2024).
