# Supplementary material for: Frustration and its impact on search and rescue canines
Source: Front Vet Sci. 2025 Mar 7;12:1546412. doi: 10.3389/fvets.2025.1546412 (PMC11926599; doi:10.3389/fvets.2025.1546412)
Supplement: Supplementary file 1 [file Data_Sheet_1.pdf]

VIRGINIA TECH

APPLIED ANIMAL BEHAVIOR & WELFARE LAB VIRGINIA TECH

Please list your first name and last name

First Name

Last Name

Dog Name

Dog breed

Dog age

Age in years

Dog sex

- Female - intact
- Female - spayed
- Male - intact
- Male - neutered
- unknown

Dogs certifications currently held or training to obtain

Do you consider your dog to be

QuestionPro

- ◯ High drive
- ◯ Medium drive
- ◯ Low drive

Does your dog wear now, or has ever worn the following collar types (check all that apply).

- ☐ Flat collar
- ☐ Choke chain
- ☐ Fur saver
- ☐ Pinch collar
- ☐ Martingale
- ☐ Nose / muzzle halter
- ☐ E-Collar
- ☐ GPS collar

For each of the 21 statements below, please place a cross in the box which most accurately describes your level of agreement with how your dog behaves in general in the range of situations listed. If the statement applies to your dog in some situations but not others, please make a judgement as to how much you agree or disagree. If your dog has never encountered the situation please mark *Not applicable* (N/A).

	5 Strongly agree	4 Mainly agree	3 Partly agree, partly disagree	2 Mainly disagree	1 Strongly disagree	N/A
1. My dog appears unsettled when there are delays in his/her routine (e.g. if walked or fed later than usual)	◯	◯	◯	◯	◯	◯
2. My dog does not like being left out of activities with other dogs	◯	◯	◯	◯	◯	◯

3. My dog is protective of his/her territory (house, garden, car)	○	○	○	○	○	○
4. When on lead my dog will persist in lunging/pulling towards something he/she would like to chase (e.g. a cat, rabbit, bird, toy)	○	○	○	○	○	○
5. My dog finds it easy to relax and settle when unable to access something he/she wants	○	○	○	○	○	○
6. My dog engages in a repetitive behavior (e.g. tail chasing, pacing, circling) when unable to access something he/she wants	○	○	○	○	○	○
7. My dog will attempt to escape if I try to confine him/her (e.g. in a room, crate or kennel)	○	○	○	○	○	○
8. My dog becomes aggressive (i.e. growl, snap or bite) if I try to remove an item he/she has (e.g. favorite toy or food)	○	○	○	○	○	○
9. My dog appears to cope well when denied access to things he/she is occasionally allowed (e.g. access to the sofa/bed or provision of table scraps)	○	○	○	○	○	○
10. I find it easy to interrupt/distract my dog from doing things he/she wants to do	○	○	○	○	○	○
	5 Strongly agree	4 Mainly agree	3 Partly agree, partly disagree	2 Mainly disagree	1 Strongly disagree	N/A
11. My dog has difficulty in responding to cues/commands (e.g. sit, lie down, stay) if there is something else he/she wants to do or access	○	○	○	○	○	○
12. My dog becomes frustrated in a large range of situations	○	○	○	○	○	○

QuestionPro

13. My dog shows increases in certain behaviors (e.g. lip licking, yawning, mounting, full body shake off) if he/she cannot immediately access something they want	○	○	○	○	○	○
14. There are days when my dog seems to become more easily frustrated than others for no apparent reason	○	○	○	○	○	○
15. When my dog is not kept busy, he/she can repeatedly lick, chew or nibble their own body parts (e.g. paws, flanks/sides)	○	○	○	○	○	○
16. My dog gets upset if shut away from visitors (e.g. vocalizes or scratches/digs at the door)	○	○	○	○	○	○
17. My dog shows continued efforts (e.g. lunging, pulling towards) to approach a dog/person they wish to greet, when being restrained from doing so (e.g. when on lead)	○	○	○	○	○	○
18. My dog appears to become frustrated frequently (e.g. at least once daily)	○	○	○	○	○	○
19. My dog becomes very excited/restless (e.g. pacing, whining, barking, jumping up) when waiting to take part in an enjoyable activity	○	○	○	○	○	○
20. My dog appears agitated and unsettled when he/she wants something another dog has (e.g. a toy or food item)	○	○	○	○	○	○
	5 Strongly agree	4 Mainly agree	3 Partly agree, partly disagree	2 Mainly disagree	1 Strongly disagree	N/A
21. My dog appears annoyed/upset if given less than he/she was expecting (e.g. wants table scrap and gets a pat on the head; given less food/a lower quality of food than expecting)	○	○	○	○	○	○

QuestionPro
